# Supplementary material for: Chitosan functionalized Mn3O4 nanoparticles counteracts ulcerative colitis in mice through modulation of cellular redox state
Source: Commun Biol. 2023 Jun 16;6:647. doi: 10.1038/s42003-023-05023-6 (PMC10275949; doi:10.1038/s42003-023-05023-6)
Supplement: Supplementary file 2 — Supplementary Information [file 42003_2023_5023_MOESM2_ESM.pdf]

Supplementary Note:

FTIR

We have also investigated proper capping and stability of Ch-Mn_3O_4 NPs through FTIR studies. Supplementary Fig. 1a showed covalent binding between C-N groups of chitosan and surface OH groups of the NPs as indicated by the perturbation of corresponding stretching bands around 1260 cm^{-1} and 1377 cm^{-1}, respectively.[1, 2]

Spectroscopic Characterization of Chitosan Capped Mn_3O_4 NPs

Further we performed detailed spectroscopic and photoluminescence study of Ch-Mn_3O_4 NPs .The absorbance spectrum of Ch-Mn_3O_4 NPs (Supplementary Fig. 2a) has several characteristic peaks which are consistent with the reported literature[1, 3, 4]. In the Supplementary Fig. S2a, two high energy absorbance bands around 280 nm and 350 nm is present. The appearance of these two high energy band is due to the charge transfer from the chitosan (i.e. ligand) to the Mn^2+/Mn^{3+} on the NP surface(i.e. metal core) and hence known as ligand to metal charge transfer (LMCT) band. Another comparatively low energy absorption band, around 430nm, is probably generated as a result of the forbidden d-d transitions of $Mn^{3+}$from $^5B_{1g}\rightarrow^5E_g$, as the degeneracy of the ^5E_g ground state term of d4 (Mn^{3+}) in a high-spin octahedral environment has been lifted by the Jahn–Teller effect. Due to the absence of alpha hydroxy-carboxylate groups in chitosan, the other possible lower energy transition from e.g., $^5B_{1g}\rightarrow^5B_{2g}$, and $^5B_{1g}\rightarrow^5A_{1g}$ were not observed [1, 3].

Three emission peaks (at UV region, blue region and green region) were observed when excited at different wavelengths (Supplementary Fig. 2b). Supplementary Fig. 2c exhibits the excitation spectra of Ch-Mn_3O_4 at their Fl. maxima that clearly indicate the direct correlation between the obtained peaks and the absorbance bands of LMCT (280 nm and 350 nm) and d-d transition (around 410 nm).

Supplementary Reference:

1. Giri A*, et al.* Rational surface modification of Mn 3 O 4 nanoparticles to induce multiple photoluminescence and room temperature ferromagnetism. *Journal of Materials Chemistry C* 1, 1885-1895 (2013).

2. Song C, Yu H, Zhang M, Yang Y, Zhang G. Physicochemical properties and antioxidant activity of chitosan from the blowfly Chrysomya megacephala larvae. *International journal of biological macromolecules* 60, 347-354 (2013).

3. Giri A*, et al.* Unprecedented catalytic activity of Mn 3 O 4 nanoparticles: potential lead of a sustainable therapeutic agent for hyperbilirubinemia. *RSC Advances* 4, 5075-5079 (2014).

4. Polley N*, et al.* Safe and symptomatic medicinal use of surface-functionalized Mn3O4 nanoparticles for hyperbilirubinemia treatment in mice. *Nanomedicine* 10, 2349-2363 (2015).

Supplementary Figures & Table

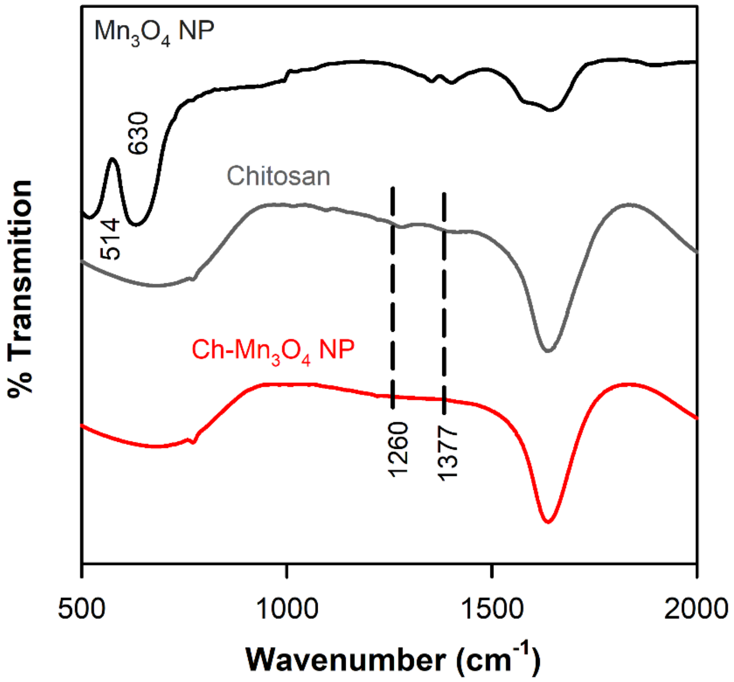

Supplementary Figure 1: FTIR Spectrum of Uncapped Mn_3O_4 NPs and Ch-Mn_3O_4 NPs.

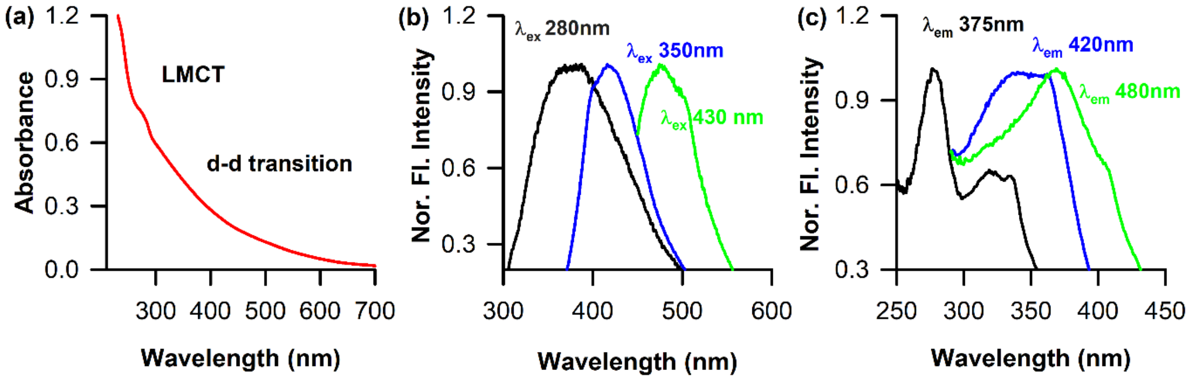

Supplementary Figure 2: (a) Absorbance Spectrum of Ch-Mn_3O_4 NPs showing LMCT and d-d transition bands. (b) Fluorescence emission spectra of Ch-Mn_3O_4 NPs. (c) Corresponding excitation spectra of Ch-Mn_3O_4 NPs.

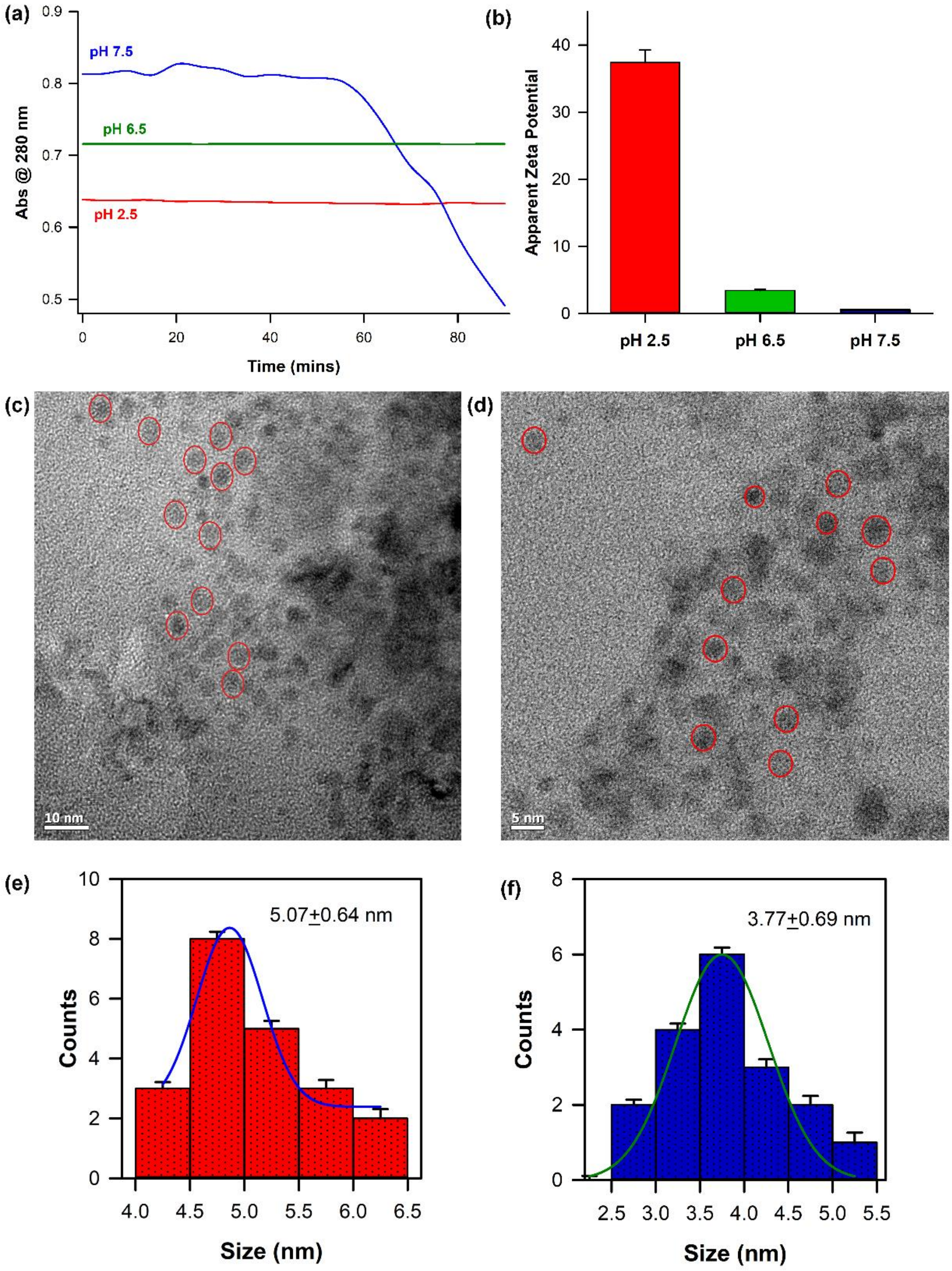

Supplementary Figure 3: Stability of Ch-Mn_3O_4 NPs. (a) Aqueous stability at different pH. (b) Zeta Potential at different pH. (c) TEM image of the NPs at pH 3. (d) TEM image of the NPs at pH 7.5. (e) Size distribution of NPs at pH 3. (f) Size distribution of NPs in pH 7.5.

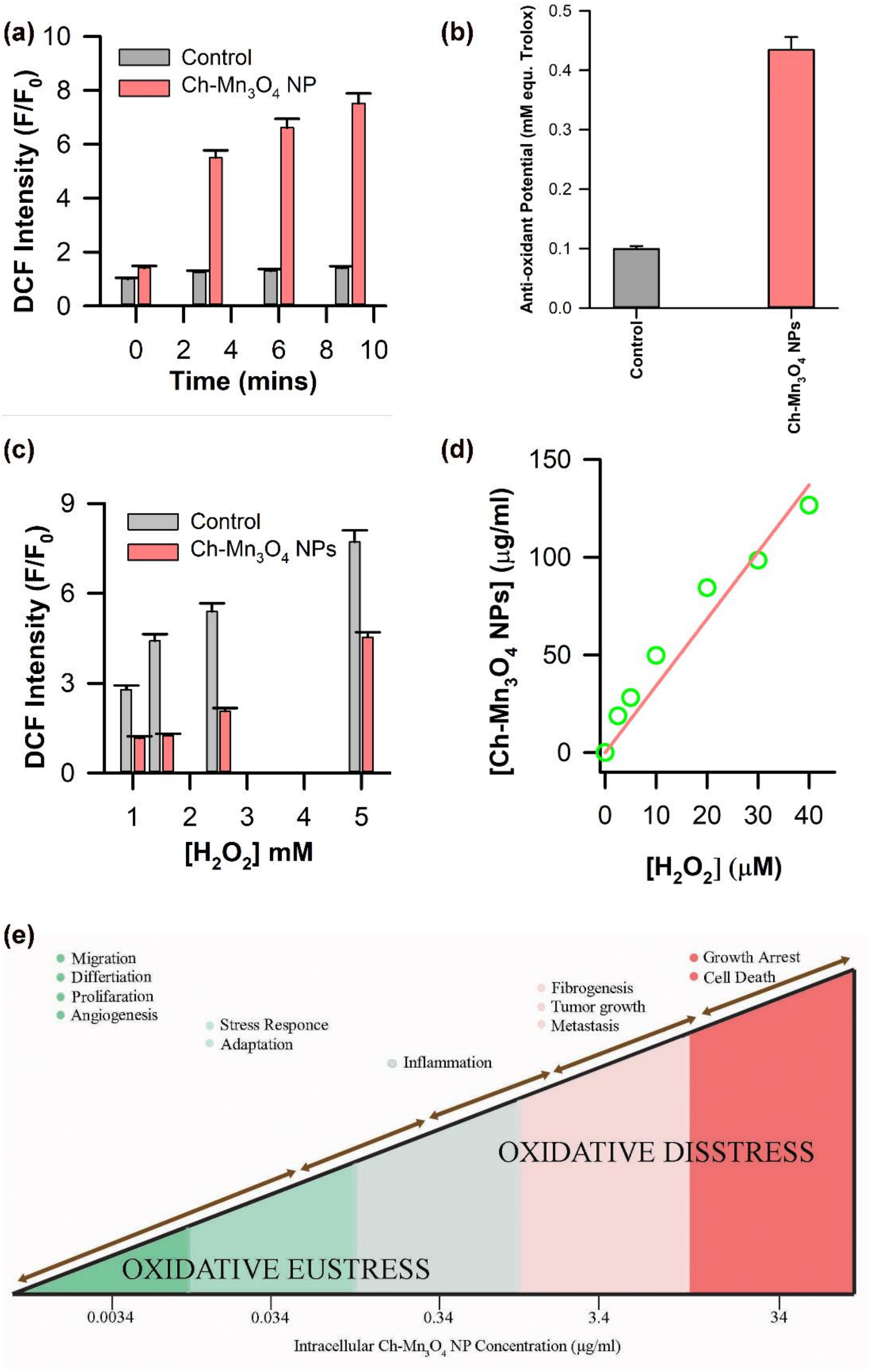

Supplementary Figure 4: (a) ROS generation by Ch-Mn_3O_4 NPs in room temperature. (b) Anti-oxidant activity of the NPs. (c) *In-vitro* redox buffering by Ch-Mn_3O_4 NPs. (d) Concentration equivalency between Ch-Mn_3O_4 NPs and H_2O_2 in term of ROS generation. (e) Relationship between concentration of Ch-Mn_3O_4 NPs and redox state of cells.

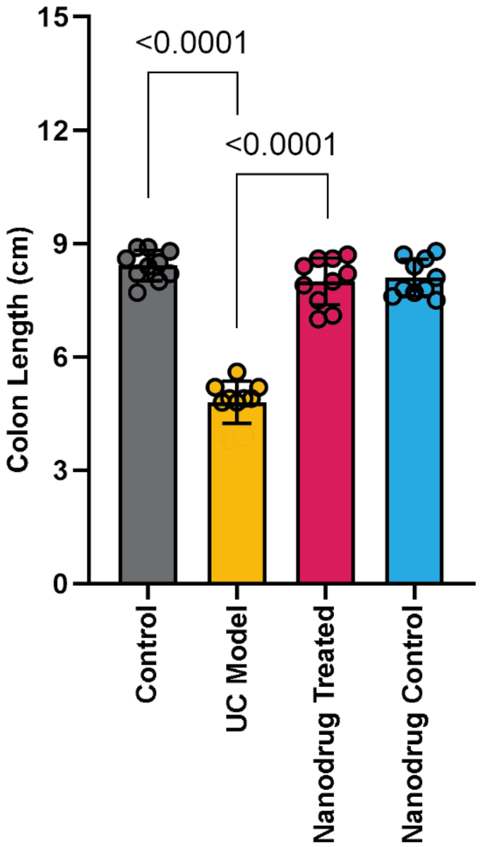

Supplementary Figure 5: Colon length of all study groups. Data are expressed as mean ± SD (n=10). Individual data points are represented as colored circles (n = 10). One-way analysis of variance (ANOVA) followed by correction of false discovery rate (post hoc FDR: two stage step up method of Benjamini, Krieger and Yekutieli) for multiple comparisons was performed for comparison between multiple groups. $p < 0.05$ is considered significant.

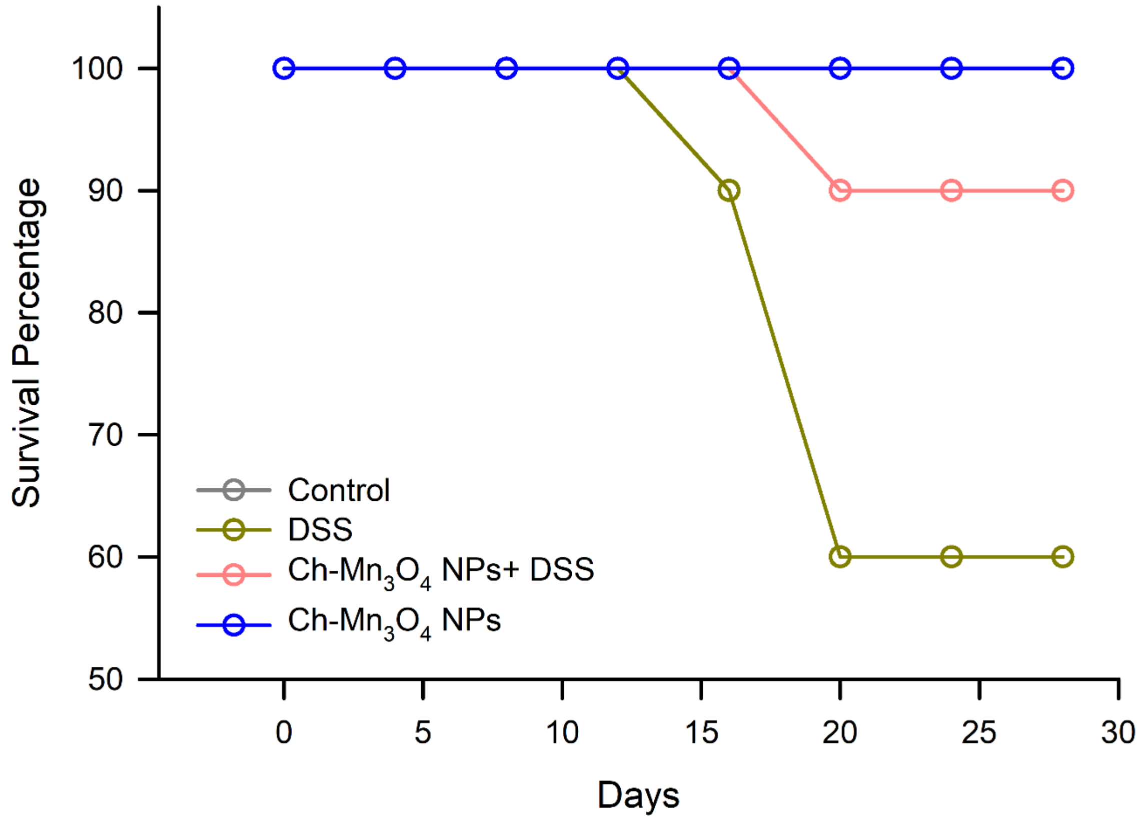

Supplementary Figure 6: Survival Curve. Survival curve of the experimental mice in all the four groups.

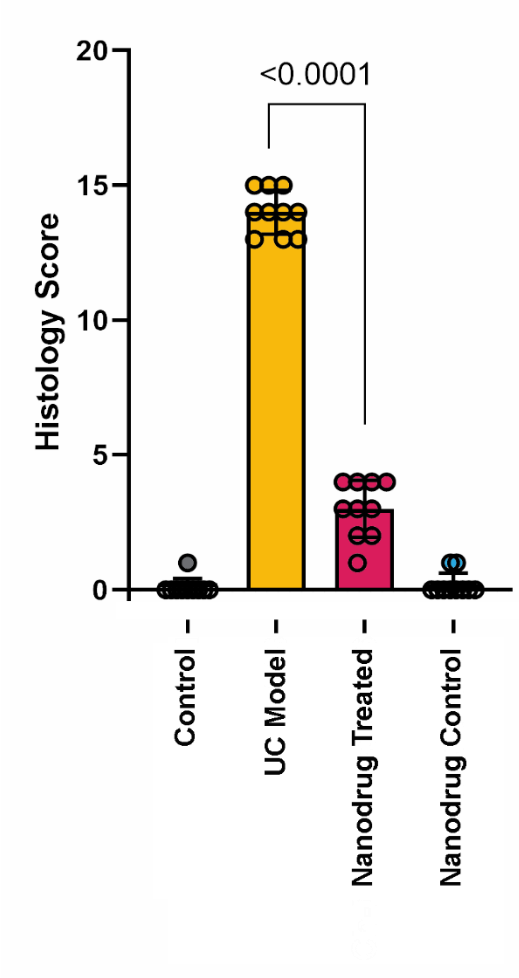

Supplementary Figure 7: Histology score of all study groups. Data are expressed as mean ± SD (n=10). Individual data points are represented as colored circles (n = 10). One-way analysis of variance (ANOVA) followed by correction of false discovery rate (post hoc FDR: two stage step up method of Benjamini, Krieger and Yekutieli) for multiple comparisons was performed for comparison between multiple groups. $p < 0.05$ is considered significant.

‘

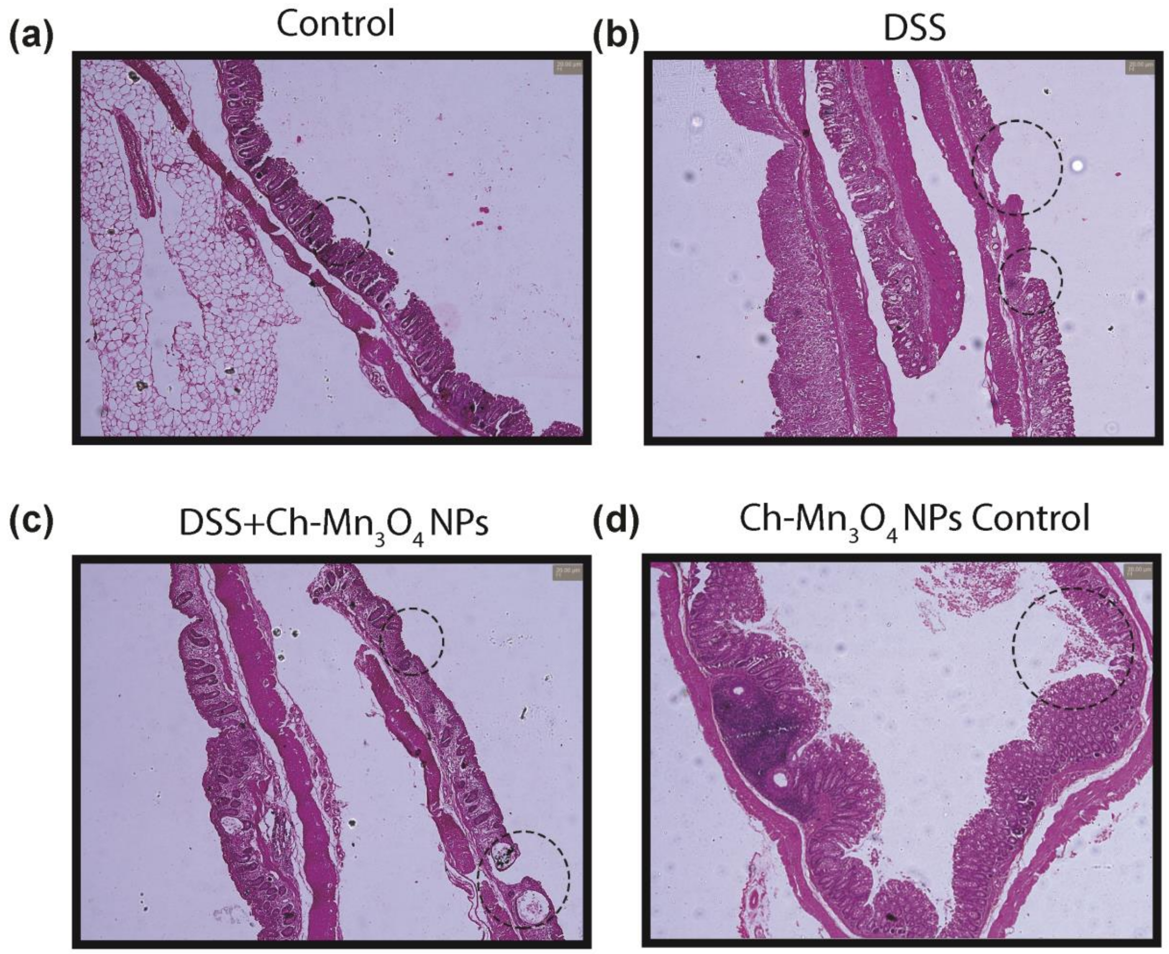

Supplementary Figure 8: Potential of Ch-Mn_3O_4 NPs in reversal of architectural changes of colon in in the animal model of ulcerative colitis. Hematoxylin and Eosin stained colon sections under microscope (Low magnification).Control: Micrographs of hematoxylin and eosin stained colon section of control mice. Black circle: Healthy mucosa. DSS: Micrographs of hematoxylin and eosin stained colon section of DSS intoxicated diseased group. Black circle: Superficial ulcer. DSS + Ch-Mn_3O_4 NPs: Micrographs of hematoxylin and eosin stained colon section of Ch-Mn_3O_4 NPs treated group. Colon section of Ch-Mn_3O_4 NPs treated mice shows the healed ulcerated area. Black circle: Healed ulcer. Ch-Mn_3O_4 NPs Control: Micrographs of hematoxylin and eosin stained colon section of Ch-Mn_3O_4 NPs control group. Black circle: Healthy mucosa.

Supplementary Table S1 (Summary of hematology parameters studied across the groups)

Parameters	Control	Ch-Mn_3O_4 NPs 1 mg kg^{-1} BW	Ch-Mn_3O_4 NPs 0.5 mg kg^{-1} BW	Ch-Mn_3O_4 NPs 0.25 mg kg^{-1} BW
**Hb (g/dl)**	13.56 ± 0.50	13.50 ±0.44	13.81 ±0.72	13.38 ±0.38
**RBC (×10^6/µl)**	6.48 ±0.48	6.44 ±0.36	6.83 ±0.41	6.44 ±0.36
**HCT (%)**	41.87±1.09	42.61 ±1.74	42.53 ±1.36	41.08 ±1.30
**MCV (fl)**	63.27 ±3.67	64.91 ±3.04	62.29 ±4.29	63.37 ±2.23
**MCH (pg)**	22.36 ±1.45	21.42 ±0.96	23.58 ±1.86	21.84 ±1.31
**MCHC (g/dl)**	34.24 ±1.16	33.31 ±1.25	33.22 ±1.09	33.72 ±1.16
**Platelets (×10^3/µl)**	6.72 ± 0.79	6.49 ± 0.52	6.64 ± 0.88	6.45 ± 1.02
**WBC (×10^5/µl)**	8.68 ± 0.55	9.72 ± 0.41	9.17 ± 0.67	8.92 ± 0.69
**Neutrophils (%)**	8.19± 1.01	8.42± 0.58	8.24± 0.65	8.63± 0.56
**Lymphoytes (%)**	76.73± 1.70	78.67± 3.10	79.71± 2.51	79.47± 3.35
**Monocytes (%)**	7.86± 0.34	7.79± 0.46	8.04± 0.75	7.71± 0.27

Hb: Haemoglobin; RBC: Red blood cell(s); HCT: Haematocrit; MCV: Mean corpuscular volume; MCH: Mean cell haemoglobin; MCHC: Mean corpuscular haemoglobin concentration; WBC: White blood cells. Data are expressed as mean ± SD (n=06). One-way analysis of variance (ANOVA) followed by correction of false discovery rate (post hoc FDR: two stage step up method of Benjamini, Krieger and Yekutieli) for multiple comparisons was performed for comparison between multiple groups.
