# Supplementary material for: Chitosan functionalized Mn3O4 nanoparticles counteracts ulcerative colitis in mice through modulation of cellular redox state
Source: Commun Biol. 2023 Jun 16;6:647. doi: 10.1038/s42003-023-05023-6 (PMC10275949; doi:10.1038/s42003-023-05023-6)
Supplement: Supplementary file 3 — Description of Additional Supplementary Files [file 42003_2023_5023_MOESM3_ESM.pdf]

Description of Additional Supplementary Files

File name: Supplementary Data 1

Description: All the raw data of main figures
