# Supplementary material for: Chitosan functionalized Mn3O4 nanoparticles counteracts ulcerative colitis in mice through modulation of cellular redox state
Source: Commun Biol. 2023 Jun 16;6:647. doi: 10.1038/s42003-023-05023-6 (PMC10275949; doi:10.1038/s42003-023-05023-6)
Supplement: Supplementary file 5 — Reporting Summary [file 42003_2023_5023_MOESM5_ESM.pdf]

nature portfolio

Corresponding author(s): Prof. Samir Kumar Pal, Prof. Saleh A. Ahmed

Last updated by author(s): Jun 5, 2023

Reporting Summary

Nature Portfolio wishes to improve the reproducibility of the work that we publish. This form provides structure for consistency and transparency in reporting. For further information on Nature Portfolio policies, see our Editorial Policies and the Editorial Policy Checklist.

Statistics

For all statistical analyses, confirm that the following items are present in the figure legend, table legend, main text, or Methods section.

n/a	Confirmed	
☐	☒	The exact sample size (*n*) for each experimental group/condition, given as a discrete number and unit of measurement
☐	☒	A statement on whether measurements were taken from distinct samples or whether the same sample was measured repeatedly
☐	☒	The statistical test(s) used AND whether they are one- or two-sided<br>*Only common tests should be described solely by name; describe more complex techniques in the Methods section.*
☒	☐	A description of all covariates tested
☐	☒	A description of any assumptions or corrections, such as tests of normality and adjustment for multiple comparisons
☐	☒	A full description of the statistical parameters including central tendency (e.g. means) or other basic estimates (e.g. regression coefficient) AND variation (e.g. standard deviation) or associated estimates of uncertainty (e.g. confidence intervals)
☐	☒	For null hypothesis testing, the test statistic (e.g. *F*, *t*, *r*) with confidence intervals, effect sizes, degrees of freedom and *P* value noted<br>*Give P values as exact values whenever suitable.*
☒	☐	For Bayesian analysis, information on the choice of priors and Markov chain Monte Carlo settings
☒	☐	For hierarchical and complex designs, identification of the appropriate level for tests and full reporting of outcomes
☒	☐	Estimates of effect sizes (e.g. Cohen's *d*, Pearson's *r*), indicating how they were calculated

Our web collection on statistics for biologists contains articles on many of the points above.

Software and code

Policy information about availability of computer code

Data collection	Data were collected using the proprietary softwares provided by the manufacturers of respective instruments detailed in the method section of the manuscript.
Data analysis	ANOVA: GraphPad Prism v8.00; Image analysis: ImageJ; Assembly of images: Adobe Photoshop CC and Illustrator CC; Data fitting: Origin Pro v8.5; Data plotting: GraphPad Prism v8.00 and SigmaPlot v14.00.

For manuscripts utilizing custom algorithms or software that are central to the research but not yet described in published literature, software must be made available to editors and reviewers. We strongly encourage code deposition in a community repository (e.g. GitHub). See the Nature Portfolio guidelines for submitting code & software for further information.

Data

Policy information about availability of data

All manuscripts must include a data availability statement. This statement should provide the following information, where applicable:

- Accession codes, unique identifiers, or web links for publicly available datasets
- A description of any restrictions on data availability
- For clinical datasets or third party data, please ensure that the statement adheres to our policy

All essential data are provided in the manuscript. Any additional data can be obtained from the corresponding author upon reasonable request.

March 2021

Human research participants

Policy information about studies involving human research participants and Sex and Gender in Research.

Reporting on sex and gender	
Population characteristics	
Recruitment	
Ethics oversight	

Note that full information on the approval of the study protocol must also be provided in the manuscript.

Field-specific reporting

Please select the one below that is the best fit for your research. If you are not sure, read the appropriate sections before making your selection.

☒ Life sciences ☐ Behavioural & social sciences ☐ Ecological, evolutionary & environmental sciences

For a reference copy of the document with all sections, see nature.com/documents/nr-reporting-summary-flat.pdf

Life sciences study design

All studies must disclose on these points even when the disclosure is negative.

Sample size	The minimum number of samples required (n=6) to get a statistically significant result in biochemical and molecular studies was chosen based on previously reported similar studies (Journal of Functional Foods. Volume 64; January 2020; 103641, Sci Rep. 2017 Feb 24;7:43126).<br><br>Number of animals in each group (N=10) was determined based on the minimum number of animals required to obtain sufficient amount of samples (e.g., blood, organ homogenate etc.) to perform biochemical and molecular studies, and recommendation of the Institutional Animal Ethics Committee (IAEC).
Data exclusions	No data were excluded.
Replication	All the experimental findings were reliably reproduced by replicating the experiments in both preclinical mice model and cellular systems.
Randomization	Experimental and control mice were randomly chosen from littermates having the correct genotype. Random samples from two experiments were selected for biochemical, histological and molecular analysis. Experiments were not randomized.
Blinding	No blinding was used during experiments or data analysis. Only, the clinical histopathologist was completely blinded to the samples (treatment groups) during histopathological observations and scoring.

Behavioural & social sciences study design

All studies must disclose on these points even when the disclosure is negative.

Study description	
Research sample	
Sampling strategy	
Data collection	
Timing	
Data exclusions	
Non-participation	
Randomization	

Ecological, evolutionary & environmental sciences study design

All studies must disclose on these points even when the disclosure is negative.

Study description	
Research sample	
Sampling strategy	
Data collection	
Timing and spatial scale	
Data exclusions	
Reproducibility	
Randomization	
Blinding	

Did the study involve field work? ☐ Yes ☐ No

Field work, collection and transport

Field conditions	
Location	
Access & import/export	
Disturbance	

Reporting for specific materials, systems and methods

We require information from authors about some types of materials, experimental systems and methods used in many studies. Here, indicate whether each material, system or method listed is relevant to your study. If you are not sure if a list item applies to your research, read the appropriate section before selecting a response.

Materials & experimental systems

n/a	Involved in the study	
☐	☒	Antibodies
☐	☒	Eukaryotic cell lines
☒	☐	Palaeontology and archaeology
☐	☒	Animals and other organisms
☒	☐	Clinical data
☒	☐	Dual use research of concern

Methods

n/a	Involved in the study	
☒	☐	ChIP-seq
☒	☐	Flow cytometry
☒	☐	MRI-based neuroimaging

Antibodies

Antibodies used	Primary antibody in IHC: Rat anti-mouse CD68 antibody, Rat anti-mouse Caspase-1 antibody, Rat anti-mouse NFκB p65 antibody (Santa Cruz Biotechnology, India). These monoclonal antibodies are cross-reactive to CD-68, Caspase-1 and NFκB p65 respectively of mouse, rat and human origin.<br><br>Secondary antibody in IHC: HRP-conjugated rabbit anti-rat secondary antibody and FITC conjugated mouse IgGFc binding antibody (Santa Cruz Biotechnology, India).
Validation	CD68: Both primary and secondary antibodies are well-validated in the literature (Primary: Nat Biomed Eng 2:810–821, 2018; J Clin

Validation

Invest 129(4):1684-1698, 2019 and Secondary: Sci Adv 7(26): eabg2517, 2021; Cell Rep 34: 108756, 2021).

Caspase1: Both primary and secondary antibodies are well-validated (Primary: Commun Biol. 2022 Feb 3;5(1):105., Commun Biol. 2021 Jun 30;4(1):824. and Secondary: Validated by the manufacturer).

NFκB p65: Both primary and secondary antibodies are well-validated (Primary: iScience 2022 May 13;25(6):104404, Proc Natl Acad Sci U S A. 2022 Aug 16;119(33):e2123097119 and Secondary: Validated by the manufacturer).

Eukaryotic cell lines

Policy information about cell lines and Sex and Gender in Research

Cell line source(s)

Human Embryonic Kidney Cells (HEK 293) were obtained from National Centre for Cell Sciences (NCCS), Maharashtra, India.

Authentication

Authentication was done by NCCS as per institutional internal quality control procedure. No further authentication was performed.

Mycoplasma contamination

Mycoplasma contamination was checked using commercially available test kit (Himedia, India) as per manufacturer's instruction.

Commonly misidentified lines (See ICLAC register)

No such misidentified cell line was used in the study.

Palaeontology and Archaeology

Specimen provenance

Specimen deposition

Dating methods

☐ Tick this box to confirm that the raw and calibrated dates are available in the paper or in Supplementary Information.

Ethics oversight

Note that full information on the approval of the study protocol must also be provided in the manuscript.

Animals and other research organisms

Policy information about studies involving animals; ARRIVE guidelines recommended for reporting animal research, and Sex and Gender in Research

Laboratory animals

Healthy 5-6 weeks old non-diabetic BALB/c mice of both sexes (50% female and 50% male) were used in the study.

Wild animals

The study did not involve wild animals.

Reporting on sex

Study findings were not related to sex specific. So, sex based data were not collected.

Field-collected samples

The study did not involve samples collected from the field.

Ethics oversight

The animal studies were performed at Central Animal Facility, Department of Zoology, Uluberia College, West Bengal, India and approved (Approval No: 03/S/UC-IAEC/01/2019)by the Institutional Animal Ethics Committee (IAEC). The standard guideline of Committee for the Control and Supervision of Experiments on Animals (CCSEA), Govt. of India, was followed.

Note that full information on the approval of the study protocol must also be provided in the manuscript.

Clinical data

Policy information about clinical studies

All manuscripts should comply with the ICMJE guidelines for publication of clinical research and a completed CONSORT checklist must be included with all submissions.

Clinical trial registration

Study protocol

Data collection

Outcomes

Dual use research of concern

Policy information about dual use research of concern

Hazards

Could the accidental, deliberate or reckless misuse of agents or technologies generated in the work, or the application of information presented in the manuscript, pose a threat to:

No	Yes	
☐	☐	Public health
☐	☐	National security
☐	☐	Crops and/or livestock
☐	☐	Ecosystems
☐	☐	Any other significant area

Experiments of concern

Does the work involve any of these experiments of concern:

No	Yes	
☐	☐	Demonstrate how to render a vaccine ineffective
☐	☐	Confer resistance to therapeutically useful antibiotics or antiviral agents
☐	☐	Enhance the virulence of a pathogen or render a nonpathogen virulent
☐	☐	Increase transmissibility of a pathogen
☐	☐	Alter the host range of a pathogen
☐	☐	Enable evasion of diagnostic/detection modalities
☐	☐	Enable the weaponization of a biological agent or toxin
☐	☐	Any other potentially harmful combination of experiments and agents

ChIP-seq

Data deposition

☐ Confirm that both raw and final processed data have been deposited in a public database such as GEO.

☐ Confirm that you have deposited or provided access to graph files (e.g. BED files) for the called peaks.

Data access links
May remain private before publication.

Files in database submission

Genome browser session
(e.g. UCSC)

Methodology

Replicates

Sequencing depth

Antibodies

Peak calling parameters

Data quality

Software

Flow Cytometry

Plots

Confirm that:

☐ The axis labels state the marker and fluorochrome used (e.g. CD4-FITC).

☐ The axis scales are clearly visible. Include numbers along axes only for bottom left plot of group (a 'group' is an analysis of identical markers).

☐ All plots are contour plots with outliers or pseudocolor plots.

☐ A numerical value for number of cells or percentage (with statistics) is provided.

Methodology

Sample preparation

Instrument

Software

Cell population abundance

Gating strategy

☐ Tick this box to confirm that a figure exemplifying the gating strategy is provided in the Supplementary Information.

Magnetic resonance imaging

Experimental design

Design type

Design specifications

Behavioral performance measures

Acquisition

Imaging type(s)

Field strength

Sequence & imaging parameters

Area of acquisition

Diffusion MRI ☐ Used ☐ Not used

Preprocessing

Preprocessing software

Normalization

Normalization template

Noise and artifact removal

Volume censoring

Statistical modeling & inference

Model type and settings

Effect(s) tested

Specify type of analysis: ☐ Whole brain ☐ ROI-based ☐ Both

Statistic type for inference
(See Eklund et al. 2016)

Correction

Models & analysis

n/a | Involved in the study
- [ ] [ ] Functional and/or effective connectivity
- [ ] [ ] Graph analysis
- [ ] [ ] Multivariate modeling or predictive analysis

Functional and/or effective connectivity

Graph analysis

Multivariate modeling and predictive analysis
